# Recent Advances in Carborane-Based Crystalline Porous Materials

**DOI:** 10.3390/molecules29163916

**Published:** 2024-08-19

**Authors:** Yuxuan Meng, Xi Lin, Jinyi Huang, Liangliang Zhang

**Affiliations:** 1 Strait Institute of Flexible Electronics (SIFE, Future Technologies), Fujian Key Laboratory of Flexible Electronics, Fujian Normal University and Strait Laboratory of Flexible Electronics (SLo-FE), Fuzhou 350017, China; qsx20221424@student.fjnu.edu.cn (Y.M.); qsz20231948@student.fjnu.edu.cn (X.L.); qsx20221429@student.fjnu.edu.cn (J.H.); 2 Frontiers Science Center for Flexible Electronics (FSCFE), MIIT Key Laboratory of Flexible Electronics (KLoFE), Northwestern Polytechnical University, Xi’an 710072, China; 3 State Key Laboratory of Structural Chemistry, Fujian Institute of Research on the Structure of Matter, Chinese Academy of Sciences, Fuzhou 350002, China

**Keywords:** carborane, crystalline porous materials, MOFs, cages, COFs

## Abstract

The field of carborane research has witnessed continuous development, leading to the construction and development of a diverse range of crystalline porous materials for various applications. Moreover, innovative synthetic approaches are expanding in this field. Since the first report of carborane-based crystalline porous materials (CCPMs) in 2007, the synthesis of carborane ligands, particularly through innovative methods, has consistently posed a significant challenge in discovering new structures of CCPMs. This paper provides a comprehensive summary of recent advances in various synthetic approaches for CCPMs, along with their applications in different domains. The primary challenges and future opportunities are expected to stimulate further multidisciplinary development in the field of CCPMs.

## 1. Introduction

The past two decades have seen the explosive growth of crystalline porous materials, including metal–organic frameworks (MOFs) and covalent organic frameworks (COFs) with typically extended networks or metal–organic cages (MOCs) and porous organic cages (POCs) with discrete structures [1]. Crystalline porous materials, constructed by coordination and covalent bonds, have exhibited various applications because of their structural tunability and modular nature. Among them, the investigation of carborane-based crystalline porous materials has attracted considerable attention due to the unique properties of carborane. These materials have special properties in light harvesting, catalysis, fluorescent sensing, biomedical applications, etc., because of the presence of boron atoms with unoccupied 2p orbitals.

MOFs are a class of crystalline porous materials composed of metal ions/clusters coordinated with organic ligands [2,3,4]. The porous structure with large surface area and well-defined pore sizes gives rise to their unique properties and potential applications [5]. As a general rule, the metal ions in MOFs can be viewed as the nodes or vertices, while the organic ligands serve as the linkers to connect metal ions and generate the final framework structure. An enormous variety of MOFs with various structures and properties can be created by combining metal ions with organic ligands, as this allows for an almost infinite number of possible combinations. What is more important, MOF properties can be customized by selecting appropriate metal ions and ligands. Because of this flexibility, MOFs with particular functions, like selective gas adsorption and separation, catalysis, drug delivery, sensing, and energy storage, can be designed and synthesized.

One of the most remarkable features of MOFs is their high porosity [6]. The framework’s pores can be precisely engineered to achieve specific dimensions and geometries, which enables the selective adsorption and containment of various gases and liquids. Such tailorability renders MOFs highly desirable for a range of applications. Furthermore, the abundance of accessible pores in MOFs endows them with an extensive surface area. This distinctive attribute positions MOFs as prime materials for catalyzing reactions. The larger surface area facilitates greater access to catalytic active sites, thereby boosting reaction efficiency and performance. Additionally, MOFs have been recognized for their applications in drug delivery systems. Their intricate porous architecture facilitates the encapsulation and precise release of pharmaceutical compounds. This feature is particularly advantageous in targeted therapies and enhances the efficacy of drug delivery processes. Furthermore, MOFs have attracted significant interest in the realms of energy storage and conversion. Extensive research continues to explore new MOFs and optimize their properties for developing innovative solutions in different industries.

MOCs are a class of supramolecular structures composed of metal ions or metal clusters coordinated with organic ligands to form three-dimensional cage-like frameworks [7,8,9]. These frameworks consist of interconnected ligand struts that surround and encapsulate the metal centers, creating an empty space or cavity inside the cage [10]. Unlike metal–organic frameworks (MOFs) that typically have extended porous structures, metal–organic cages are discrete molecular entities with well-defined shapes and sizes. This characteristic allows for precise control over their geometry, porosity, and functionality, making them ideal for various applications, including sensors, switches, and stimulus-responsive systems.

COFs are a class of porous materials composed of covalently linked organic building blocks [11,12]. They are different from MOFs in that the backbone of COFs consists entirely of organic molecules instead of metal ions. Due to the regular arrangement of the building blocks, COFs are characterized by their distinct structures and large surface areas. Thus, the strong covalent bonds connecting the organic units ensure excellent stability of these materials. Since their chemical structure can be precisely controlled, the size, shape, and functionality of the pores can be customized, making COFs adaptable to a wide range of uses [13]. One of the most significant advantages of COFs is their tunable porosity. The design and synthesis of COFs can be customized to create cavities and channels of various sizes, enabling selective adsorption and separation of molecules. This property is valuable in applications such as gas storage and separation. COFs also show promise in catalysis due to their well-defined active sites and high surface area. The porosity and chemical nature of COFs can be tailored to accommodate different catalytic species, allowing for enhanced reactivity and selectivity in various reactions. Additionally, COFs have been explored for applications in electronic and optoelectronic devices. Their well-ordered structures and the possibility of introducing organic chromophores and π-conjugated systems into the framework make them attractive for applications in areas such as sensors, photovoltaics, and light-emitting devices. The development of COFs is a rapidly growing field, and researchers continue to explore new synthetic methods, structural designs, and applications. The combination of their crystalline structure, tunable porosity, and diverse functionalities allows for a wide range of possibilities in areas.

Carboranes are carbon–boron molecular clusters which can be viewed as three-dimensional analogues to benzene [14]. They are finding many applications in medicine, materials, and organometallic chemistry [15]. Thus, their exceptional thermal and chemical stabilities, as well as 3D structures, make them very difficult to functionalize, in particular the regioselective functionalization of the B-H vertex among ten similar B-H bonds [16,17].

**Scheme 1:**
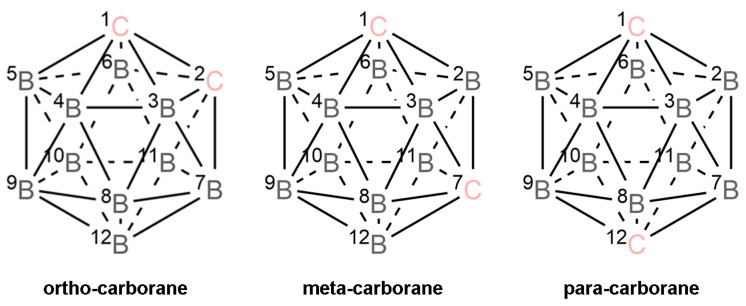
Structures of *ortho-/meta-/para-*carborane (in the figure, take dicarbon carborane with 12 vertices as an example).

Carboranes are icosahedral clusters comprising carbon and boron (Scheme 1), known for their advantageous properties such as rigidity, thermal stability, and chemical inertness [16,17]. Dicarbon carboranes, represented by the formula C_2_B_n−2_H_n_ (with 6 ≤ n ≤ 12), exhibit three-dimensional delocalized aromaticity, wherein surface and core bonding correspond to σ- and π-bonding, respectively. These molecular structures demonstrate promising potential in various material applications due to their unique electronic and structural properties [18].

**Scheme 2:**
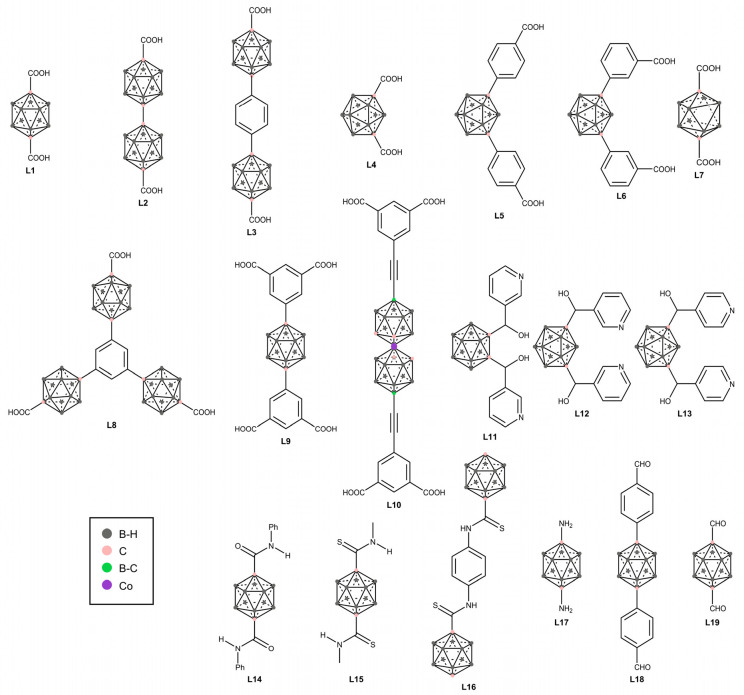
The representative carborane ligands used to construct CCPMs.

This review presents recent advancements of carborane-based crystalline porous materials (CCPMs) and differs from previous reviews that have summarized carborane in other fields in that it focuses on methods for the integration of carborane ligands (Scheme 2) into crystalline porous materials like MOFs, MOCs, and COFs, which offers various advantages. This paper is to review the design strategies of carborane-based crystalline porous materials from a broad perspective, including (i) the introduction of carborane into the building blocks to accomplish the assembly of the porous structure, (ii) selectively activating the B-H bond to achieve the directional coordination self-driven assembly with non-covalent forces, and (iii) introducing carborane, which is already present in the structure, to the side groups by post-synthetic modification, etc. In addition, the potential applications of carborane-based crystalline porous materials in fields such as molecular sieving, energy storage, catalysis, and medicine are also discussed. It is our hope that this review will serve as a valuable reference for those interested in the application of carborane in crystalline porous materials.

## 2. Carborane-Based Metal–Organic Frameworks

Carborane-based MOFs are a special type of MOF that incorporate carborane clusters into their structures. The incorporation of carboranes enhances their hydrophobicity, chemical inertness, and thermal stability. These properties make carborane-based MOFs suitable for applications such as gas storage, separation, and catalysis under harsh conditions.

The carborane clusters can serve as redox-active sites, allowing them to store and transfer charge effectively. This characteristic makes them potential candidates for the development of high-performance supercapacitors and advanced batteries. Carborane-based MOFs represent a new generation of MOFs that combine the unique properties of carboranes with the versatility of MOF frameworks. These materials have diverse applications and hold great potential for various fields, including energy, catalysis, and medicine. Ongoing research aims to further understand and optimize their properties to unlock their full potential.

### 2.1. Carboxylate-Based Carborane MOFs

**Figure 1:**
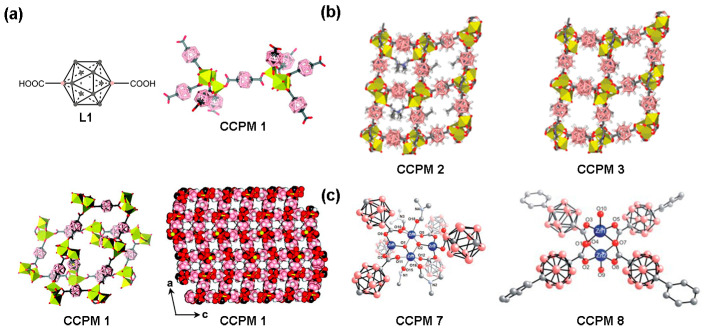
(**a**) Structure of CCPM **1**. Reproduced with permission from ref. [18]. Copyright © 2007, American Chemical Society. (**b**) Structure of CCPMs **2–3**. Reproduced with permission from ref. [19]. Copyright © 2008 Royal Society of Chemistry. (**c**) Structure of CCPMs **7–8**. Reproduced with permission from ref. [22]. Copyright © 2010 Elsevier.

In 2007, Mirkin and Hupp opened up new possibilities to construct boron-rich MOFs by utilizing carborane as building blocks for the first time. Solvothermal reactions of 1,12-dihydroxy-carbonyl-1,12-dicarba-*closo*-dodecaborane (**L1**) with Zn(NO_3_)·6H_2_O yielded CCPM **1** (Figure 1a). It should be pointed out that the structure of CCPM **1** was different from that of MOF-5. The carboxylate ligands adopt monodentate bridging mode to connect octahedral Zn ions instead of forming Zn_4_O clusters in MOF-5. Because of the introduction of the rigidity of dicarborane, the DEF-free version of CCPM **1** showed exceptionally high H_2_ uptake at 77 K [18]. Subsequently, based on the success of H_2_ uptake, the previous carborane-based MOFs were used to separate and purify mixtures of CO_2_ and CH_4_ at 298 K. Both experiments and IAST calculations demonstrated that CCPMs **2** and **3** (Figure 1b) with open metal sites were promising materials for the separation and purification of (quadru)polar/nonpolar pairs [19]. In order to certify the essential role of the solvothermal reaction in the synthesis of carborane-based MOFs, Mirkin and Hupp et al. synthesized three cobalt(II)-carborane-based MOFs with different morphologies [20]. The adsorption capabilities and selectivities of CCPMs **4** and **5** were highly dependent upon the structural differences, activation conditions, and resulting pore structure [21].

In addition to commonly used carboxylic acid ligands, the boron cage can also serve as an organic ligand to construct MOFs through nonclassic B-H···M interactions. Zhang and Xing reported a variety of boron cage pillared supramolecular MOFs based on the *closo*-dodecaborate cluster [B_12_H_12_]^2-^, which achieved high-performance separation of light hydrocarbons [23,24,25,26,27,28,29,30]. As a derivative of the [B_12_H_12_]^2-^ cluster, neutral *para*-carborane (*p*-C_2_B_10_H_12_) (**L1**) can also be used to build MOFs, which exhibit abundant weakly polarized effects for the selective adsorption and separation of light hydrocarbons. Recently, Zhang’s group reported the first case of a carborane cage pillared MOF (CCPM **6**) with pcu topology. The presence of carborane not only increases the stability towards water but also reverses the adsorption sequence of C_2_H_6_ over C_2_H_4_, which was further confirmed by through a combination of in situ gas-loaded single-crystal structures and density functional theory (DFT) calculations. Study on the absorption mechanism showed that there were stronger Van der Waals interactions and C^δ-^-H^δ+^···H^δ+^-B^δ-^ dihydrogen interactions between C_2_H_6_ and poorly polarized channels of MOFs. A high-purity C_2_H_4_ with a production of approximately 14.5 L/kg can be obtained from one-step C_2_H_4_/C_2_H_6_ mixtures [31]. Compared with their reports on [B_12_H_12_] ^2−^ pillared MOFs, the hydrophobic carborane hybrid MOF displayed reversed C_2_H_6_ adsorption properties due to the specific C-H^δ+^···H^δ-^-B dihydrogen bonds and other Van der Waals interactions [25,26,28].

The carborane-based MOFs that were previously discussed all demonstrated exceptionally high thermal stability. In some cases, the frameworks maintained their stability up to 510 °C, which is significantly higher than the majority of the MOFs [18]. However, constrained by the steric hindrance of *p*-carborane with three-dimensional delocalized aromatic systems, a close structural analogue of MOF-5 could not be obtained, which severely influenced the pore volumes and internal surfaces of carborane-based MOFs. Apparently, utilizing the relatively long carborane ligands offers an excellent solution wherever porosity problems are a concern. The Hupp and Mirkin group reported another two microporous MOFs based on longer carborane carboxylate-based ligands (**L2** and **L3**) by reaction with Zn(NO_3_)·6H_2_O (Figure 1c). The BET surface areas of CCPMs **7** and **8** can reach up to 1180 and 800 m^2^/g, respectively, resulting in structures with greater porosity [22].

Besides d-block metal ions such as Zn^II^ and Co^II^, lanthanide ions holding versatile coordination geometry and high coordination numbers can also be used to assemble porous networks, even though the Ln cation coordination spheres and target materials have particular properties that are more challenging to regulate. Furthermore, compared with transition-metal ions, lanthanide ions with higher coordination numbers can accommodate more coordinatively unsaturated and Lewis acidic sites upon removal of coordinated guest molecules, benefiting gas storage and separation.

**Figure 2:**
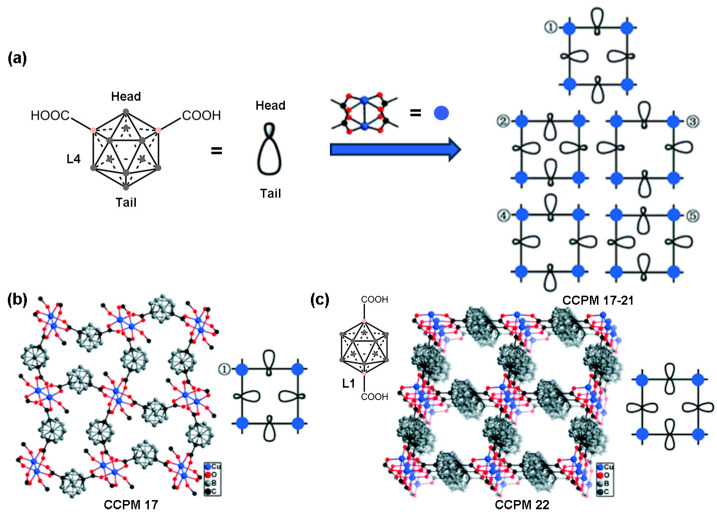
(**a**) The formation of 2D structures with five types of rings, (**b**) the structure of CCPM 17, and (**c**) the structure of CCPM 22. Reproduced with permission from ref. [33]. Copyright © 2012 Royal Society of Chemistry.

Based on the above considerations, Jin’s group designed and synthesized a series of microporous lanthanide MOFs containing *p*-dicarboxylate carborane ligands (**L1**) with three distinct structure types effected by lanthanide contraction (CCPMs **9–16**). Significantly, because of the presence of coordinatively unsaturated metal centers, the carborane- and Ln-based MOFs showed outstanding selective gas adsorption properties for CO_2_ over CH_4_ and N_2_ at room temperature [32]. Subsequently, Jin and coauthor expanded their research to bent *m*-CDC^2-^ (**L4**) and Cu_2_(CO_2_)_4_ paddle-wheel units according to their experience in controlled B-H activation. It is a wonder that the solvent molecules play an important role in the assembly of the five types of rings with a head and a tail in the 2D grid structures (CCPMs **17–21**) (Figure 2). They are different from the bent *m*-CDC^2-^ ligand in that only one structure type could be obtained when using the linear *p*-CDC^2-^ ligand (**L1**) without a head and a tail (CCPM **22**) (Figure 2c) [33,34].

In fact, carboranes can be regarded as three-dimensional delocalized aromatic systems with abundant σ-bonding and π-bonding, which endow the corresponding carborane-based materials with rigidity, thermal stability, and chemical stability. Thus, carborane compounds possess excellent hydrophobicity, because of the presence of polarized B-H bonds and spherical geometry and hydrophobic surface. Motivated by the remarkable qualities of carborane MOFs, Stylianou and Planas developed a novel mixed-linker *meta*-carborane-based MOF with extended *m*-CDC^2-^ ligands (**L5**), coordinated DABCO ligands, and a Cu_2_(CO_2_)_4_ paddle-wheel. The resulting *meta*-carborane-based MOF (CCPM **23**) was resistant to severe basic and acidic aqueous conditions without losing its porosity. Thus, both vapor-phase dynamic adsorption breakthrough experiments and Monte Carlo and DFT calculations revealed that CCPM **23** was an excellent candidate for biobutanol recovery from a mostly water-containing ABE mixture [35]. Moreover, CCPM **23** presented better performance than that of MOF-74(Ni) towards adsorptive selectivity for CO_2_ and N_2_ mixtures [36,37]. After that, the same group reported two examples of carborane-based Cu_2_ paddle-wheel MOFs (CCPMs **24** and **25**) for increasing hydrolytic stability by rational design of symmetrical and unsymmetric carborane-based dicarboxylic linkers (**L5** and **L6**) [38,39]. In addition, the heterogeneous catalysis activities of carborane-based Cu_2_ paddle-wheel MOFs towards the aza-Michael reaction were also investigated. In their continuing exploration of multifunction carborane-based MOFs, they paid attention to water-stable carborane-based Ln MOFs (CCPMs **26** and **27**). As expected, a series of carborane-based Ln MOFs were fabricated with high stability in a broad range of pH values (3–11). Thus, the resulting Ln MOFs presented the typical emission feature of Ln ions, together with the sensitization effect of carborane-based ligands (**L5**) as antenna molecules. The ET process of mixed-metal Ln-MOFs was also modulated by controlling energy transfer (ET) efficiency between Tb^3+^ and Eu^3+^. The high thermal and water stability, excellent luminescence properties, and proneness to spray-coating ensure their potential application in the field of anticounterfeiting as security inks [40].

**Figure 3:**
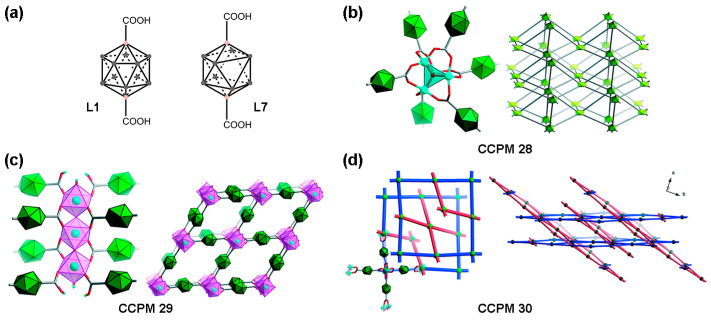
(**a**) The carborane ligands of L1 and L7 and (**b**–**d**) the structure of CCPMs 28–30. Reproduced with permission from ref. [41]. Copyright © 2016 Royal Society of Chemistry.

Some initial efforts were directed towards the assembly of 12-vertex carborane ligands and metal ions. De Vos and Baše fabricated zinc, cobalt, and copper porous MOFs (CCPMs **28–30**) (Figure 3) based on a smaller 10-vertex closo-carborane ligand (**L7**) analogue for the first time [41]. They assumed that the strain differences between ligands would affect the physisorption. Though they did not synthesize an MOF-5 analogue based on terephthalic acid, the resulting three MOFs were highly porous and robust with distinctive topologies. Their experiments also demonstrated that even slight steric truncation of ligands could be of crucial importance to the final structure during self-assembly. After that, the 10- and 12-vertex *p*-bicarboranedicarboxylic acids (**L1** and **L7**) serve as the organic linkers in the construction of these MOFs, with cobalt ions acting as the metal nodes (CCPMs **31–34**). The differences in the vertex numbers (10 and 12) lead to distinct structural topologies and, consequently, different gas adsorptive properties, highlighting their potential for applications such as gas storage and separation [42].

Very recently, Macreadie and Farha compared the effect of increasing linker dimensionality on the flexibility and pore geometry. On the one hand, the introduction of 3D aromatic linkers would decrease the pore sizes, which was a benefit for sieving separation. On the other hand, the rigidity and stability would be increased, enabling them to be used in separation procedures on an industrial scale. As excepted, NU-2004 (CCPM **35**), constructed by a carborane-based 3D linker and aluminum and nodes, showed a significant improvement in hexane isomer separation when compared to the parent 2DL MOF, MIL-53, and 3DL MOF, NU-2000 [43].

As a benefit from the development of coordination chemistry and reticular chemistry, a variety of tritopic and tetratopic carboxylic ligands based on carborane have been designed and synthesized for MOF construction. The incorporation of tritopic and tetratopic carborane carboxylic ligands into MOFs might endow not only abundant topological structures but also promising porosities and storage capacities. For example, Mirkin fabricated the first MOF (CCPM **36**) based on a tritopic carborane ligand (**L8**) with a BET surface area of 1870 m^2^ g^−1^. In contrast to the analogous MOF-143 based on H_3_BTB, both porosity and stability have been significantly improved [44]. Similar situations can be derived from their continuous investigation on carborane MOFs (CCPMs **37** and **38**) based on tetratopic carboxylic linkers (**L9** and **L10**) [45,46].

### 2.2. Pyridine-Based Carborane MOFs

As is well known, the coordination bond between metals and ligands is a crucial factor determining the stability of MOFs. Therefore, constructing stable MOFs can be achieved by enhancing the strength of the metal–ligand coordination bond. According to the hard and soft acids and bases (HSABs) theory, low-valent transition metal ions act as soft acids and can form stable MOFs when coordinated with soft bases containing N-heterocyclic ligands, such as pyridine, imidazole, pyrazole, triazole, tetrazole, and so on [47,48]. Thus, N-heterocyclic ligands typically have higher p*K*a values than carboxylic acid ligands and exhibit strong affinity towards low-valent transition metal ions. This enables the MOFs synthesized using N-heterocyclic ligands to remain stable in water/alkaline solutions [49]. Therefore, as an important branch, N-containing MOFs have also attracted more attention in the fields of materials science and chemistry.

**Figure 4:**
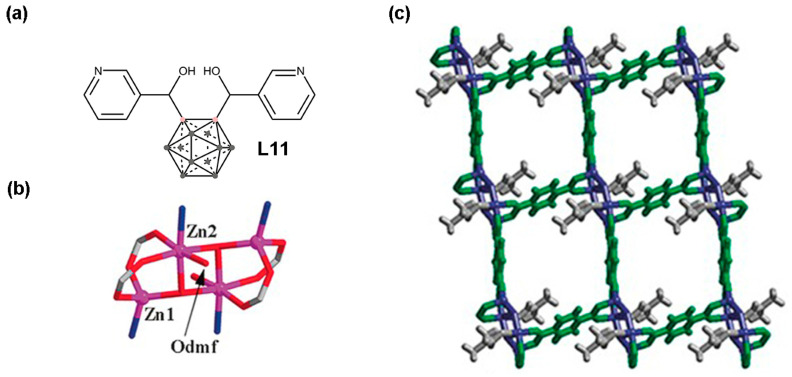
(**a**) The L11 linker, (**b**) Zn_4_(O)_2_(OOC)_4_ cluster, and (**c**) CCPM 39 layers. Reproduced with permission from ref. [51]. Copyright © 2016 Wiley-VCH Verlag GmbH & Co. KGaA, Weinheim, Germany.

A series of disubstituted carboranyl alcohol derivatives bearing nitroaromatic rings have been designed and synthesized to investigate their chiral recognition [50]. The supramolecular O−H···N, together with O−H···O hydrogen bonds, dominated the crystal packings of these o-carboranyl alcohols. Furthermore, Maspoch and Planas developed a mixed-linker Zn-MOF (CCPM **39**) (Figure 4) based on H_2_bdc and 1,2-bis{(pyridin-3-yl)methanol}-1,2-dicarba-closo-dodecarborane (oCB-L) (**L11**). The functionalized ortho-carborane organic linkers with pyridylmethylalcohol groups at the C-positions endow MOFs with switchable properties between hydrophobic and superhydrophilic. It is interesting that the switching behavior was reversible at least two cycles by gradually eliminating **L11** on the crystal surface in NaOH and bdc linkers in H_2_O. When the **L11** were exposed to the outer surfaces and inner pores of MOFs, they were hydrophobic, when the bdc linkers appeared on the outer surface, they turned superhydrophilic. The present work may boost the development of smart MOF materials, especially in the field of stimulus-responsive materials based on carborane [51].

Inspired by the intriguing properties of N-heterocyclic carborane-based MOFs, the same group unveiled the first research on their flexibility and unexpected dynamic behavior after two years. The 3D porous MOF (CCPM **40** and **41**), as a kind of third-generation MOF, was constructed by flexible dipyridyl-based carborane linkers (**L12**) and rigid Co/BTB layers [52]. Subsequently, the same group paid attention to disubstituted m-carboranylpyridylalcohols and reported six transition-metal MOFs (CCPM **42–47**) based on closo-dodecaboranes (**L12** and **L13**) and polycarboxylates ligands. The synthetic, structural chemistry and guest-dependent mechanical properties were intensively studied [53].

With the development of synthetic and structural chemistry, the post-synthetic modification was considered a powerful tool to enrich the structures and performance of N-heterocyclic carborane-based MOFs. In order to overcome the low conductivity and improve electrochemical performance of MOFs, Planas et al. synthesized MOF and polypyrrole composites using a post-synthetic modification strategy. Compared with the mother MOF (CCPM **40**), both the stability and electrochemical performance were highly enhanced [54].

## 3. Carborane-Based Metal–Organic Cages

Carborane-based MOCs are an intriguing subclass of MOCs that integrate carborane clusters into their structures. Carboranes, with their unique geometry and properties, offer a distinctive building block for constructing such cages. Thus, these carborane-based MOCs exhibit several noteworthy characteristics. Firstly, they inherit the exceptional thermal and chemical stability of carboranes, which can enhance the overall robustness of the cage structure. Additionally, the presence of carboranes can impart hydrophobicity and chemical inertness to the MOCs, expanding their potential applications in areas such as gas storage, catalysis, and molecular recognition. The incorporation of carborane clusters into MOCs also introduces intriguing electronic and redox properties. Carboranes can serve as redox-active sites within the cage framework, enabling applications in electron transfer processes and electrocatalysis. Moreover, the tunability of carborane-based MOCs offers opportunities for fine-tuning their properties and functionalities through structural modifications. This tunability enables tailored applications in diverse fields. Carborane-based metal–organic cages represent a promising avenue in the realm of supramolecular chemistry, combining the unique attributes of carboranes with the versatility of metal–organic frameworks.

### 3.1. Steric-Effect-Directed B–H Bond Activation of Carboranes

The distinctive three-dimensional arrangement of carborane clusters, coupled with their numerous weak intermolecular forces, makes them highly effective building blocks for supramolecular assembly. Over the last few years, extensive research has been dedicated to tackling the complexities of functionalizing carborane clusters and activating B-H bonds selectively. For instance, the coordination-driven self-assembly technique has been applied to facilitate the formation of metallacycles from carborane structures. This is achieved through the direct interaction of carborane with metal ions, leading to the creation of these novel metallacycles [55].

In fact, the controlled activation of B-H bonds in *para*-carboranes is more complex than that in *ortho*- and *meta*-carboranes owing to the uniform chemical environment of the B-H bonds, which makes selective activation more difficult. It is possible to achieve single-site activation, but subsequent activations may lead to the formation of multiple isomers at various sites, such as B(2,3), B(2,4), B(2,7), B(2,8), and B(2,9).

**Figure 5:**
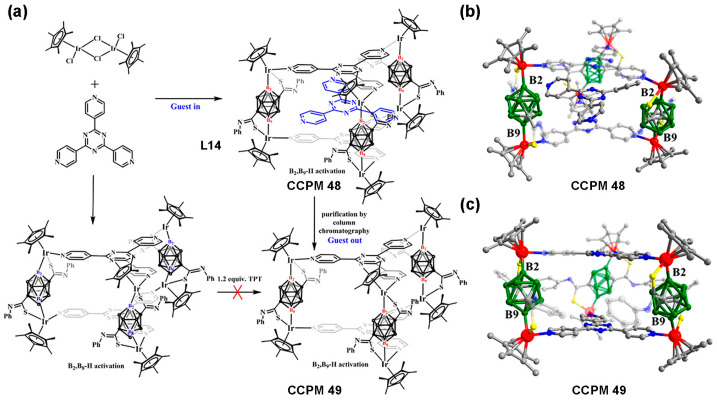
(**a**) The synthesis of CCPM 48 and 49 based on L14. Crystallographically derived molecular structures of CCPM 48 (**b**) and CCPM 49 (**c**) (the H atoms and OTf– ions have been omitted for clarity). Color code: Ir, red; S, yellow; N, blue; C, gray; B, dark green. Reproduced with permission from refs. [56,57]. Copyright © 2021, American Chemical Society.

Recently, Jin developed a directed-coordination-guided noncovalent-force-based cooperative self-assembly strategy to modulate the B-H bond activation of *para*-carboranes. It is reported that the π–π interactions play an important role in activating B-H bond selectively with the choice of linear pyridine linkers. Activation patterns such as B(2,8)-H or B(2,7)-H were attained. Remarkably, through the utilization of host–guest interactions in metallacage compounds, the activation of B(2,8)-H bonds could be circumvented, leading to exclusive activation of B(2,9)-H bonds. When the tritopic ligand 2,4,6-tri(4-pyridyl)-1,3,5-triazine (TPT) was employed, the carborane-based metallacycle transformed to a metal–organic cage (CCPM **48**) (Figure 5b) based on B(2,8)-H bond activation. It is interesting that an excess equivalent of TPT ligands gives rise to an intermediate species (CCPM **49**) (Figure 5c), corresponding to one TPT molecule trapped into cages with B(2,9)-H bond activation. The incorporation of the guest molecule serves as a kind of template to shorten the distance and increase the π–π interactions. Thus, a carborane-based metal–organic cage was obtained with B(2,8)-H and B(2,9)-H activation, due to the steric hindrance effects in TTF-Py ligands [56].

It is a great challenge to separate light hydrocarbons (C_1_–C_9_), such as benzene and cyclohexane, because of their similar boiling points. The electrostatic driving forces of carborane may offer a platform for encapsulating guest molecules through dihydrogen bond interactions (B^δ+^-H^δ-^···H^δ+^-C^δ-^), π–π interactions, and so on. Jin and co-authors reported a carborane-based metal–organic cage (CCPM **50**) with B(2,9)-H activation, exhibiting high selective separation ability toward benzene and cyclohexane [58].

### 3.2. “Cage Walking”

**Figure 6:**
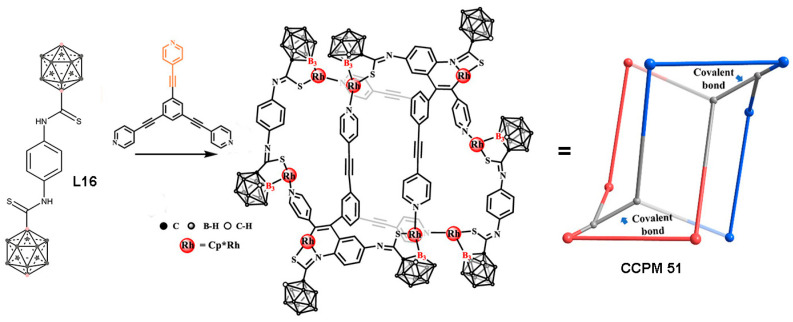
Schematic representation of the synthesis of CCPM 51 and the simplified diagram of CCPM 51, which can be viewed as a ravel composed of interlocks linked by covalent bonds. Reproduced with permission from ref. [57]. Copyright © 2023 American Chemical Society.

Recently, in order to construct unusual unsymmetrical supramolecular cages, the same group revealed a novel strategy by integrating the B-C coupling reaction and “cage walking” process. Specifically, when bipyridine linkers with acetylene were used, carborane-based metal–organic cages were formed, along with a B-C coupling reaction, and metallization site transformed from B3 to B4 in *o*-carborane, which indicates the occurrence of “cage walking”. Furthermore, when tridentate-pyridine linkers were used, a kind of carborane-based metal–organic cage (CCPM **51**) (Figure 6) with a novel ravel structure was formed, along with M-C and C-C couplings. This work likely contributes to the advancement of the field of carborane-based metal–organic cages by expanding the range of possible structures and properties that can be achieved through the careful selection of linkers and reaction conditions [57].

## 4. Carborane-Based Covalent Organic Frameworks

Carborane-based covalent organic frameworks represent a fascinating intersection of carborane chemistry and porous organic materials. The integration of carborane units into COFs offers several advantages. Firstly, carboranes possess high thermal and chemical stability, which can enhance the overall stability of the COF structure. This stability is particularly advantageous for applications requiring robust materials, such as gas storage, catalysis, and separation processes.

Additionally, carborane-based COFs can exhibit enhanced hydrophobicity and chemical inertness due to the presence of the carborane moieties. This property makes them attractive for applications in environments where moisture or chemical reactivity is a concern.

Furthermore, carborane units can introduce unique electronic and optical properties to COFs, thereby expanding their potential applications in areas such as optoelectronics, photonics, and sensing. The electron-rich nature of carboranes can facilitate charge transfer processes within the COF framework, leading to tunable electronic conductivity and redox activity.

The modular nature of COF synthesis allows for the precise control of pore size, surface area, and chemical functionality, offering opportunities for tailoring the properties of carborane-based COFs for specific applications. By judiciously choosing the organic linkers and optimizing synthetic conditions, researchers can design carborane-containing COFs with desired pore architectures and properties.

A carborane cage is polyhedral carbon-containing boron cluster, having a hydrophobic B–H surface and dihydrogen bond (B–H^δ−^···H^δ+^–C), which could be used in the organometallic field and materials science [59,60]. And carborane has been regarded as a boron carrier, which is electron deficient. Carborane-based COFs with high crystallinity, excellent physicochemical properties, and good porosity show potential applications in adsorption separation, molecular sieving, drug delivery systems, energy storage, and energy transformation.

### 4.1. Host–Guest Interactions

**Figure 7:**
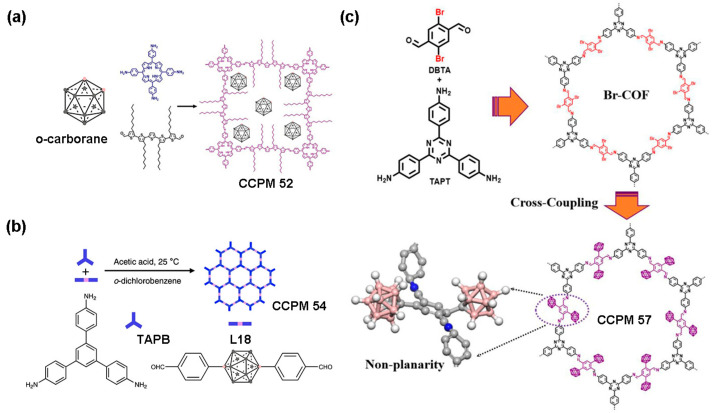
(**a**) Synthesis of the structure of CCPM 52. Reproduced with permission from ref. [61]. Copyright © 2020, American Chemical Society. (**b**) Synthesis of the structure of CCPM 54. Reproduced with permission from ref. [62]. Copyright © 2023 Springer Nature. (**c**) Synthetic route toward the CCPM 57 via cross-coupling. Reproduced with permission from ref. [63]. Copyright © 2023 American Chemical Society.

Because of the tunable porosity and excellent structure stability of COFs, carborane can be easily immobilized into their structure. Liu et al. demonstrated a one-pot embedding strategy to encapsulate carborane into COFs (DSPE-BCOP-5T) (CCPM **52**) (Figure 7a) [61]. They prepared a kind of carborane-loaded nanoscale COF, which is tailored in size by alkyl-chain engineering to accomplish excellent pharmacokinetics, achieving the abundant accumulation of boron-10 in tumor cells. CCPM **52** was synthesized from TAPP (15.0 mg, 0.022 mmol), o-carborane (31.72 mg, 0.22 mmol), and DF5T (40.4 mg, 0.044 mmol) in a solvent mixture of n-BuOH (1.0 mL), o-DCB (1.0 mL), and acetic acid (3 M, 0.1 mL) via the Schiff base condensation reaction. The porous structure of CCPM **52** was utilized to encapsulate the carborane and the surface of the COF was modified with 1,2-distearoyl-sn-glycero-3-phosphoethanolamine-N-[amino(polyethylene-glycol)-2000] (DSPE-PEG) to form stable aqueous-phase nanoparticles for enhanced stability during drug delivery and to improve the efficiency and accuracy of drug delivery. Incorporating octyl chains onto thiophene on the imine-linked to porphyrin achieves a suitable size of COF in pharmacokinetics in vivo. On the other hand, this modification weakens the interlayer π–π interactions through steric hindrance, resulting in good solubility and dispersibility in solvents. This carborane-loaded COF can not only be used for multifunctionality in cancer diagnosis and therapy but also overcomes the drawbacks of conventional small-molecule BNCT drugs.

### 4.2. Acting as Building Blocks

It is well known that new types of COFs with desired characteristics can be constructed by combining different building blocks and that building blocks can largely determine the topological structures, physicochemical properties, and functionalities of COFs. The introduction of carborane as a building block is one of the approaches to synthesize carborane-based COFs.

#### 4.2.1. Two-Dimensional COFs

In 2D COFs, the atomic layers with well-defined alignment of π architectural units, which are further stacked to crystallize laminar structures of π-skeletons through π–π interactions, provide the fundamental basis for fully controllable structural designs, including the sizes, shapes, and environments of 1D channels [64]. Yu et al. synthesized an amphiphilic carborane-based COF (CCPM **53**) and treated it as a nano-trapper for polysulfides [65]. The combination of plane-symmetric triple-connected knots and linear-symmetric di-connected linkers leads to the formation of 2D hexagonal COFs. CCPM **53** was synthesized from **L17** (11 mg, 0.07 mol) and 1,3,5-triformylphloroglycinol (10 mg, 0.05 mol) via the Schiff base condensation reaction. Boron-rich cluster compounds formed using partial substitution of boron atoms by carbon atoms in borane. Boron-rich carborane are highly electron deficient and are expected to have a strong affinity with electron-rich polysulfides. The lithophilic and thiophilic groups in the carborane based COFs can provide multiple adsorption sites for directly capture of polysulfides, thus improving the chemical affinity and capture efficiency for polysulfides, which efficiently suppresses the “shuttle effect,” leading to a high-rate capacity (314 mA h g^–1^ after 1000 cycles at 2.5 C) and an ultra-long cycling life (after 1000 cycles with a very low decay rate of 0.0395% per cycle at 1 C) of LSBs under high sulfur loading.

In addition, Liu et al. developed a carborane-based COF (CCPM **54**) (Figure 7b) as a boron “capsule” of immune adjuvants for simultaneous boron neutron capture therapy (BNCT) and immunotherapy [62]. CCPM **54** with high crystallinity were synthesized from 1,3,5-tris (4-aminophenyl) benzene (TAPB) (7 mg, 0.02 mmol) and *p*-carborane-1,10-phenyl-dialdehyde (**L18**) (10.3 mg, 0.03 mmol) by Schiff base condensation. To passively adsorb imiquimod with CCPM **54** in ethanol, the imiquimod-loaded CCPM **54** was purified by centrifugation and washed with dimethyl sulfoxide (DMSO) and ethanol. To synthesize the boron “capsule”, the prepared imiquimod-loaded CCPM **54** was modified with the DSPE-PEG2000. After a freeze-drying process, boron capsules were finally obtained. The neutron-activated boron capsule was designed and prepared to synergize the localized nuclear reaction of BNCT and trigger controlled-release of immune adjuvants to stimulate an effective anti-tumor immune response.

#### 4.2.2. Three-Dimensional COFs

**Figure 8:**
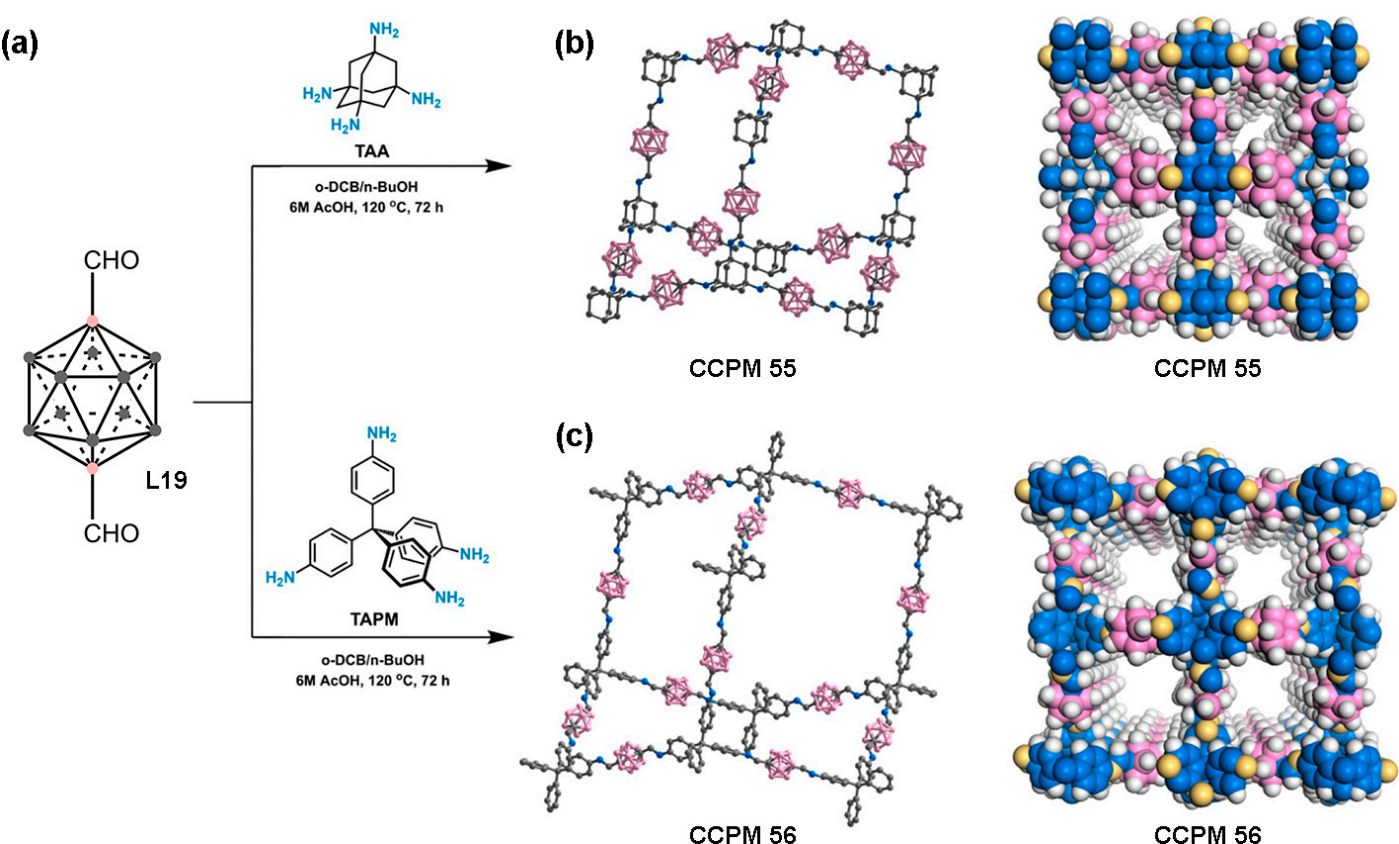
(**a**) Schematic diagrams for the synthesis of CCPM 55 and CCPM 56 under solvothermal conditions and structure of (**b**) CCPM 55 and (**c**) CCPM 56 (C, blue spot; N, yellow spot; B, pink spot; H, white spot). Reproduced with permission from ref. [66]. Copyright © 2023 Royal Society of Chemistry.

The geometries of the building blocks control the development of the elementary polygon skeletons in a 2D or 3D manner, leading to the generation of 2D or 3D COFs. Compared with the layered 2D COFs, 3D COFs are less common owing to the limited diversity of tetrahedron-type knots [64]. Huang et al. attempted to construct 3D crystal-structured COFs, exploiting the interactions of the two hydrogen bonds of carborane, B-H and C-H, to develop their adsorption and to isolate the alkane isomers [66]. CCPM **55** and **56** (Figure 8) were synthesized by combining linear diformyl-*p*-carborane (**L19**) with 1,3,5,7-tetraaminoadamantane (TAA) and tetrahedral tetra (4-aminophenyl) methane (TAPM), respectively. TAA-DFCB-COF and TAPM-DFCB-COF were obtained as white solids, as analogues of COF-300, and CCPM **55** and **56** were expected to have a 3D dia-net interpenetration structure with concrete channel diameters of 0.6 and 1.0 nm, respectively. Compared with layered 2D COFs, 3D COFs have the outstanding advantages of high specific surface area, complex structure, and complete accessibility of active sites. They developed a novel functional COF by integrating carborane-based units into a 3D dia-net architecture. The obtained COFs are unique in that they have interpenetrating channels enclosed by dense carborane, which possess high affinity with alkanes via the (B-H^δ−^···H^δ+^-C) dihydrogen bond interactions. The carborane-based COFs have a seven-fold interpenetrated *dia*-net structure with a large surface area and high chemical stability. This work provided a potentially versatile platform for the development of carborane-based COFs, which offers a new facet for designing functional COFs as high-performance adsorbents.

### 4.3. Post-Synthetic Modification

The presence of ordered and well-defined pores in COFs opens the possibility of incorporating pendant groups which can undergo functional group interconversion. This strategy, commonly known as pore-wall engineering or channel-wall modification, is a very versatile approach to introduce new functionalities into COFs [67]. Post-synthetic modification can improve the crystallinity of COFs to some degree and introduce the expected functions to expand the applications of carborane-modified COFs. Yu et al. proposed a post-synthesis modification (PSM) strategy for the synthesis of fibrous-carborane-tailored covalent organic frameworks (CCPM **55**) (Figure 7c) serving as a barrier to polysulfide to inhibit the shuttle effect [63]. Br-COFs were synthesized by 2,5-dibromoterephthaldehyde (DBTA) and 2,4,6-tris (4-aminophenyl)-1,3,5-triazine (TAPT) via Schiff base condensation. Then, CCPM **57** was synthesized through the cross-coupling of carboranyllithium (1-Li-1,2-C_2_B_10_H_11_) with bromide ligands. CCPM **57** has the advantages of amphiphilicity, and the boron clusters of o-Carborane building blocks could efficiently improve the chemical sorption of LiPSs. The microporous channels of PMCB-COF could facilitate the transport of lithium ions and the triazine groups can form Li bonds with lithium ions as dipole–dipole interactions. CCPM **57** provides plentiful active sites (C = N and B–H) and large porosity for the sorption of LiPSs. The LSBs assembled with a CCPM **57** separator could deliver a reversible capacity as high as 926 mAh g^–1^ at 1 C. In addition, the capacity decays only 0.039% for each cycle (sulfur content of 80% in the cathode).

## 5. Conclusions and Perspectives

In conclusion, the classifications and design strategies of carborane-based crystalline porous materials were reviewed in detail. The reported research outcomes have demonstrated that the carborane-based crystalline porous materials have attracted considerable attention due to possessing superior hydrophobicity, thermal stability, and chemical inertness. It indicates that carborane-based crystalline porous materials are more suitable for applications in the fields of energy storage or catalysis under certain harsh conditions. This paper provides an overview of the frontiers of development of carbaborane-based crystalline porous materials by reviewing the reported works with a particular focus on the fields of MOFs, MOCs, and COFs.

Although carborane-based crystalline porous materials have received increasing attention in recent years, they are still at a stage where many aspects remain unexplored. Since the research on carborane-based MOCs and carborane-based COFs started slightly later, the reported works have not yet reached a mature stage compared to those regarding carborane-based MOFs. In comparison to the extensive coordination mode of MOFs, the methods for loading carborane onto cages or COFs is relatively limited. Consequently, future research could focus on enriching the structure of carboranes as a building blocks to synthesize COFs and cages with different spatial configurations, such as exploring the potential application of carborane as tetrahedral-type nodes.

In terms of loading carborane for constructing carborane-based crystalline porous materials, the examples of synthesizing crystalline porous materials by building blocks within carborane. And carborane also acts as a ligand to directly pillar the crystalline porous materials. Or selectively activating the B-H bond to achieve directional coordination self-driven assembly with non-covalent forces. And carborane can be used as a guest and be coated in crystalline porous materials as well. And introduces carborane to the synthesized crystalline porous materials by post-synthetic modification, etc., are presented. All of these synthetic strategies have been successfully employed for the preparation of various carborane-based crystalline porous materials, resulting in the optimization of a multitude of physicochemical properties of the crystalline porous materials. Additionally, these synthetic strategies have provided guidance for the synthesis of other unexplored carborane-based crystalline porous materials. Furthermore, the advantageous properties of hydrophobicity, thermal stability, and chemical inertness in carborane-based crystalline porous materials have prompted scientists to make great efforts to investigate their diverse potential applications, including the controlled release of drugs, energy storage, energy conversion, sieving of molecules, gas adsorption, catalysis, and so forth. Although these works cover a broad range of applications of carborane-based crystalline porous materials, research on their practical applications lacks depth and systematicity. And there is still a need to expand the functionality and potential applications of carborane-based COFs and cages in different fields compared to carborane-based MOFs.

The potential of carborane-based crystalline porous materials in industrial production is considerable, and it is hoped that researchers interested in carborane-based crystalline porous materials will be inspired by this review to propose further novel ideas that will facilitate the emergence of new materials and applications in this field. Therefore, it will bring about a qualitative leap in the development of these materials.

## Data Availability

No new data were created or analyzed in this study. Data sharing is not applicable to this article.

## References

[1] El-Sayed E.-S.M., Yuan Y.D., Zhao D., Yuan D. (2022). Zirconium Metal–Organic Cages: Synthesis and Applications. Acc. Chem. Res..

[2] Furukawa H., Cordova K.E., O’Keeffe M., Yaghi O.M. (2013). The Chemistry and Applications of Metal-Organic Frameworks. Science.

[3] Zhou H.-C., Long J.R., Yaghi O.M. (2012). Introduction to Metal–Organic Frameworks. Chem. Rev..

[4] Xiao W., Cheng M., Liu Y., Wang J., Zhang G., Wei Z., Li L., Du L., Wang G., Liu H. (2023). Functional Metal/Carbon Composites Derived from Metal–Organic Frameworks: Insight into Structures, Properties, Performances, and Mechanisms. ACS Catal..

[5] Yang L., Qian S., Wang X., Cui X., Chen B., Xing H. (2020). Energy-efficient separation alternatives: Metal–organic frameworks and membranes for hydrocarbon separation. Chem. Soc. Rev..

[6] Gagliardi L., Yaghi O.M. (2023). Three Future Directions for Metal–Organic Frameworks. Chem. Mater..

[7] Lin H., Xiao Z., Le K.N., Yan T.h., Cai P., Yang Y., Day G.S., Drake H.F., Xie H., Bose R. (2022). Assembling Phenothiazine into a Porous Coordination Cage to Improve Its Photocatalytic Efficiency for Organic Transformations. Angew. Chem. Int. Ed..

[8] Gosselin A.J., Rowland C.A., Bloch E.D. (2020). Permanently Microporous Metal–Organic Polyhedra. Chem. Rev..

[9] Fang Y., Murase T., Sato S., Fujita M. (2013). Noncovalent Tailoring of the Binding Pocket of Self-Assembled Cages by Remote Bulky Ancillary Groups. J. Am. Chem. Soc..

[10] Vardhan H., Yusubov M., Verpoort F. (2016). Self-assembled metal–organic polyhedra: An overview of various applications. Coord. Chem. Rev..

[11] Ding S.-Y., Gao J., Wang Q., Zhang Y., Song W.-G., Su C.-Y., Wang W. (2011). Construction of Covalent Organic Framework for Catalysis: Pd/COF-LZU1 in Suzuki–Miyaura Coupling Reaction. J. Am. Chem. Soc..

[12] Tan K.T., Ghosh S., Wang Z., Wen F., Rodríguez-San-Miguel D., Feng J., Huang N., Wang W., Zamora F., Feng X. (2023). Covalent organic frameworks. Nat. Rev. Methods Primers.

[13] Huang N., Zhai L., Coupry D.E., Addicoat M.A., Okushita K., Nishimura K., Heine T., Jiang D. (2016). Multiple-component covalent organic frameworks. Nat. Commun..

[14] King R.B. (2023). Trivalent Polyhedra as Duals of Borane Deltahedra: From Molecular Endohedral Germanium Clusters to the Smallest Fullerenes. Molecules.

[15] Avdeeva V.V., Nikiforova S.E., Malinina E.A., Sivaev I.B., Kuznetsov N.T. (2023). Composites and Materials Prepared from Boron Cluster Anions and Carboranes. Materials.

[16] Cheng R., Qiu Z., Xie Z. (2017). Iridium-catalysed regioselective borylation of carboranes via direct B–H activation. Nat. Commun..

[17] Chen M., Xu J., Zhao D., Sun F., Tian S., Tu D., Lu C., Yan H. (2022). Site-Selective Functionalization of Carboranes at the Electron-Rich Boron Vertex: Photocatalytic B−C Coupling via a Carboranyl Cage Radical. Angew. Chem. Int. Ed..

[18] Farha O.K., Spokoyny A.M., Mulfort K.L., Hawthorne M.F., Mirkin C.A., Hupp J.T. (2007). Synthesis and Hydrogen Sorption Properties of Carborane Based Metal−Organic Framework Materials. J. Am. Chem. Soc..

[19] Bae Y.-S., Farha O.K., Spokoyny A.M., Mirkin C.A., Hupp J.T., Snurr R.Q. (2008). Carborane-based metal–organic frameworks as highly selective sorbents for CO_2_ over methane. Chem. Commun..

[20] Farha O.K., Spokoyny A.M., Mulfort K.L., Galli S., Hupp J.T., Mirkin C.A. (2009). Gas-Sorption Properties of Cobalt(II)–Carborane-Based Coordination Polymers as a Function of Morphology. Small.

[21] Bae Y.-S., Spokoyny A.M., Farha O.K., Snurr R.Q., Hupp J.T., Mirkin C.A. (2010). Separation of gas mixtures using Co(ii) carborane-based porous coordination polymers. Chem. Commun..

[22] Spokoyny A.M., Farha O.K., Mulfort K.L., Hupp J.T., Mirkin C.A. (2010). Porosity tuning of carborane-based metal–organic frameworks (MOFs) via coordination chemistry and ligand design. Inorg. Chim. Acta.

[23] Zhang Y., Yang L., Wang L., Cui X., Xing H. (2019). Pillar iodination in functional boron cage hybrid supramolecular frameworks for high performance separation of light hydrocarbons. J. Mater. Chem. A.

[24] Zhang Y., Yang L., Wang L., Duttwyler S., Xing H. (2019). A Microporous Metal-Organic Framework Supramolecularly Assembled from a Cu(II) Dodecaborate Cluster Complex for Selective Gas Separation. Angew. Chem..

[25] Zhang Y., Hu J., Krishna R., Wang L., Yang L., Cui X., Duttwyler S., Xing H. (2020). Rational Design of Microporous MOFs with Anionic Boron Cluster Functionality and Cooperative Dihydrogen Binding Sites for Highly Selective Capture of Acetylene. Angew. Chem. Int. Ed..

[26] Zhang Y., Wang L., Hu J., Duttwyler S., Cui X., Xing H. (2020). Solvent-dependent supramolecular self-assembly of boron cage pillared metal–organic frameworks for selective gas separation. CrystEngComm.

[27] Wang L., Sun W., Duttwyler S., Zhang Y. (2021). Efficient adsorption separation of methane from CO_2_ and C2–C3 hydrocarbons in a microporous closo-dodecaborate [B_12_H_12_]^2-^ pillared metal–organic framework. J. Solid State Chem..

[28] Wang L., Sun W., Zhang Y., Xu N., Krishna R., Hu J., Jiang Y., He Y., Xing H. (2021). Interpenetration Symmetry Control Within Ultramicroporous Robust Boron Cluster Hybrid MOFs for Benchmark Purification of Acetylene from Carbon Dioxide. Angew. Chem. Int. Ed..

[29] Sun W., Hu J., Duttwyler S., Wang L., Krishna R., Zhang Y. (2022). Highly selective gas separation by two isostructural boron cluster pillared MOFs. Sep. Purif. Technol..

[30] Sun W., Jin Y., Wu Y., Lou W., Yuan Y., Duttwyler S., Wang L., Zhang Y. (2022). A new boron cluster anion pillared metal organic framework with ligand inclusion and its selective acetylene capture properties. Inorg. Chem. Front..

[31] Wang L., Wu S., Hu J., Jiang Y., Li J., Hu Y., Han Y., Ben T., Chen B., Zhang Y. (2024). A novel hydrophobic carborane-hybrid microporous material for reversed C_2_H_6_ adsorption and efficient C_2_H_4_/C_2_H_6_ separation under humid conditions. Chem. Sci..

[32] Huang S.L., Lin Y.J., Yu W.B., Jin G.X. (2012). Porous Frameworks Based on Carborane–Ln_2_(CO_2_)_6_: Architecture Influenced by Lanthanide Contraction and Selective CO_2_ Capture. ChemPlusChem.

[33] Huang S.-L., Weng L.-H., Jin G.-X. (2012). Bottom-up synthesis of coordination polymers based on carborane backbones and Cu_2_(CO_2_)_4_ paddle-wheel: Ligand metathesis with metallotecons. Dalton Trans..

[34] Wang X., Guo Q., Kong T. (2015). Tetraethylenepentamine-modified MCM-41/silica gel with hierarchical mesoporous structure for CO_2_ capture. Chem. Eng. J..

[35] Gan L., Chidambaram A., Fonquernie P.G., Light M.E., Choquesillo-Lazarte D., Huang H., Solano E., Fraile J., Viñas C., Teixidor F. (2020). A Highly Water-Stable meta-Carborane-Based Copper Metal–Organic Framework for Efficient High-Temperature Butanol Separation. J. Am. Chem. Soc..

[36] Gan L., Andres-Garcia E., Mínguez Espallargas G., Planas J.G. (2023). Adsorptive Separation of CO_2_ by a Hydrophobic Carborane-Based Metal–Organic Framework under Humid Conditions. ACS Appl. Mater. Interfaces.

[37] Wang X., Zeng W., Song M., Wang F., Hu X., Guo Q., Liu Y. (2019). Polyetheramine improves the CO_2_ adsorption behavior of tetraethylenepentamine-functionalized sorbents. Chem. Eng. J..

[38] Li Z., Choquesillo-Lazarte D., Fraile J., Viñas C., Teixidor F., Planas J.G. (2022). Rational design of carborane-based Cu^2-^paddle wheel coordination polymers for increased hydrolytic stability. Dalton Trans..

[39] Gan L., Fonquernie P.G., Light M.E., Norjmaa G., Ujaque G., Choquesillo-Lazarte D., Fraile J., Teixidor F., Viñas C., Planas J.G. (2019). A Reversible Phase Transition of 2D Coordination Layers by B–H∙∙∙Cu(II) Interactions in a Coordination Polymer. Molecules.

[40] Li Z., Núñez R., Light M.E., Ruiz E., Teixidor F., Viñas C., Ruiz-Molina D., Roscini C., Planas J.G. (2022). Water-Stable Carborane-Based Eu^3+^/Tb^3+^ Metal–Organic Frameworks for Tunable Time-Dependent Emission Color and Their Application in Anticounterfeiting Bar-Coding. Chem. Mater..

[41] Boldog I., Bereciartua P.J., Bulánek R., Kučeráková M., Tomandlová M., Dušek M., Macháček J., De Vos D., Baše T. (2016). 10-Vertex closo-carborane: A unique ligand platform for porous coordination polymers. CrystEngComm.

[42] Boldog I., Dušek M., Jelínek T., Švec P., Ramos F.S.d.O., Růžička A., Bulánek R. (2018). Porous 10- and 12-vertex (bi)-p-dicarba-closo-boranedicarboxylates of cobalt and their gas adsorptive properties. Microporous Mesoporous Mater..

[43] Idrees K.B., Kirlikovali K.O., Setter C., Xie H., Brand H., Lal B., Sha F., Smoljan C.S., Wang X., Islamoglu T. (2023). Robust Carborane-Based Metal–Organic Frameworks for Hexane Separation. J. Am. Chem. Soc..

[44] Clingerman D.J., Morris W., Mondloch J.E., Kennedy R.D., Sarjeant A.A., Stern C., Hupp J.T., Farha O.K., Mirkin C.A. (2015). Stabilization of a highly porous metal–organic framework utilizing a carborane-based linker. Chem. Commun..

[45] Kennedy R.D., Krungleviciute V., Clingerman D.J., Mondloch J.E., Peng Y., Wilmer C.E., Sarjeant A.A., Snurr R.Q., Hupp J.T., Yildirim T. (2013). Carborane-Based Metal–Organic Framework with High Methane and Hydrogen Storage Capacities. Chem. Mater..

[46] Kennedy R.D., Clingerman D.J., Morris W., Wilmer C.E., Sarjeant A.A., Stern C.L., O’Keeffe M., Snurr R.Q., Hupp J.T., Farha O.K. (2014). Metallacarborane-Based Metal–Organic Framework with a Complex Topology. Cryst. Growth Des..

[47] Yuan S., Feng L., Wang K., Pang J., Bosch M., Lollar C., Sun Y., Qin J., Yang X., Zhang P. (2018). Stable Metal–Organic Frameworks: Design, Synthesis, and Applications. Adv. Mater..

[48] Feng L., Wang K.-Y., Day G.S., Ryder M.R., Zhou H.-C. (2020). Destruction of Metal–Organic Frameworks: Positive and Negative Aspects of Stability and Lability. Chem. Rev..

[49] Ding M., Cai X., Jiang H.-L. (2019). Improving MOF stability: Approaches and applications. Chem. Sci..

[50] Di Salvo F., Paterakis C., Tsang M.Y., García Y., Viñas C., Teixidor F., Giner Planas J., Light M.E., Hursthouse M.B., Choquesillo-Lazarte D. (2013). Synthesis and Crystallographic Studies of Disubstituted Carboranyl Alcohol Derivatives: Prevailing Chiral Recognition?. Cryst. Growth Des..

[51] Rodríguez-Hermida S., Tsang M.Y., Vignatti C., Stylianou K.C., Guillerm V., Pérez-Carvajal J., Teixidor F., Viñas C., Choquesillo-Lazarte D., Verdugo-Escamilla C. (2016). Switchable Surface Hydrophobicity–Hydrophilicity of a Metal–Organic Framework. Angew. Chem., Int. Ed..

[52] Tan F., López-Periago A., Light M.E., Cirera J., Ruiz E., Borrás A., Teixidor F., Viñas C., Domingo C., Planas J.G. (2018). An Unprecedented Stimuli-Controlled Single-Crystal Reversible Phase Transition of a Metal–Organic Framework and Its Application to a Novel Method of Guest Encapsulation. Adv. Mater..

[53] Tsang M.Y., Rodríguez-Hermida S., Stylianou K.C., Tan F., Negi D., Teixidor F., Viñas C., Choquesillo-Lazarte D., Verdugo-Escamilla C., Guerrero M. (2017). Carborane Bis-pyridylalcohols as Linkers for Coordination Polymers: Synthesis, Crystal Structures, and Guest-Framework Dependent Mechanical Properties. Cryst. Growth Des..

[54] Li Z., Fraile J., Viñas C., Teixidor F., Planas J.G. (2021). Post-synthetic modification of a highly flexible 3D soft porous metal–organic framework by incorporating conducting polypyrrole: Enhanced MOF stability and capacitance as an electrode material. Chem. Commun..

[55] Cui P.-F., Liu X.-R., Jin G.-X. (2023). Supramolecular Architectures Bearing Half-Sandwich Iridium- or Rhodium-Based Carboranes: Design, Synthesis, and Applications. J. Am. Chem. Soc..

[56] Cui P.-F., Liu X.-R., Guo S.-T., Lin Y.-J., Jin G.-X. (2021). Steric-Effects-Directed B–H Bond Activation of para-Carboranes. J. Am. Chem. Soc..

[57] Liu X.-R., Cui P.-F., Guo S.-T., Lin Y.-J., Jin G.-X. (2023). “Cage Walking” Synthetic Strategy for Unusual Unsymmetrical Supramolecular Cages. J. Am. Chem. Soc..

[58] Cui P.-F., Liu X.-R., Lin Y.-J., Li Z.-H., Jin G.-X. (2022). Highly Selective Separation of Benzene and Cyclohexane in a Spatially Confined Carborane Metallacage. J. Am. Chem. Soc..

[59] Cui P.-F., Lin Y.-J., Li Z.-H., Jin G.-X. (2020). Dihydrogen Bond Interaction Induced Separation of Hexane Isomers by Self-Assembled Carborane Metallacycles. J. Am. Chem. Soc..

[60] Fujii S., Masuno H., Taoda Y., Kano A., Wongmayura A., Nakabayashi M., Ito N., Shimizu M., Kawachi E., Hirano T. (2011). Boron Cluster-based Development of Potent Nonsecosteroidal Vitamin D Receptor Ligands: Direct Observation of Hydrophobic Interaction between Protein Surface and Carborane. J. Am. Chem. Soc..

[61] Shi Y., Fu Q., Li J., Liu H., Zhang Z., Liu T., Liu Z. (2020). Covalent Organic Polymer as a Carborane Carrier for Imaging-Facilitated Boron Neutron Capture Therapy. ACS Appl. Mater. Interfaces.

[62] Shi Y., Guo Z., Fu Q., Shen X., Zhang Z., Sun W., Wang J., Sun J., Zhang Z., Liu T. (2023). Localized nuclear reaction breaks boron drug capsules loaded with immune adjuvants for cancer immunotherapy. Nat. Commun..

[63] Li M., Yu J., Xue Y., Wang K., Wang Q., Xie Z., Wang L., Yang Y., Wu J., Qiu X. (2023). Preparation of Carborane-Tailored Covalent Organic Frameworks by a Postsynthetic Modification Strategy as a Barrier to Polysulfide in Lithium–Sulfur Batteries. ACS Appl. Mater. Interfaces.

[64] Huang N., Wang P., Jiang D. (2016). Covalent organic frameworks: A materials platform for structural and functional designs. Nat. Rev. Mater..

[65] Zhu Y., Yang J., Qiu X., Li M., He G., Wang Q., Xie Z., Li X., Yu H. (2021). Amphiphilic Carborane-Based Covalent Organic Frameworks as Efficient Polysulfide Nano-Trappers for Lithium–Sulfur Batteries. ACS Appl. Mater. Interfaces.

[66] Xu X., Cui Q., Chen H., Huang N. (2023). Carborane-Based Three-Dimensional Covalent Organic Frameworks. J. Am. Chem. Soc..

[67] Segura J.L., Royuela S., Mar Ramos M. (2019). Post-synthetic modification of covalent organic frameworks. Chem. Soc. Rev..

